# Hybrid Lipid Nanocapsules: A Robust Platform for mRNA Delivery

**DOI:** 10.1021/acsami.4c00992

**Published:** 2024-03-20

**Authors:** Sunil Kumar Yadava, B. Pradeep Kumar Reddy, Mark R. Prausnitz, Marcus T. Cicerone

**Affiliations:** † School of Chemistry and Biochemistry, Georgia Institute of Technology, Atlanta, Georgia 30332, United States; ‡ School of Chemical and Biomolecular Engineering, Georgia Institute of Technology, Atlanta, Georgia 30332, United States

**Keywords:** hybrid lipid nanocapsules (hLNCs), mRNA delivery, firefly luciferase, nanoparticles, vaccine

## Abstract

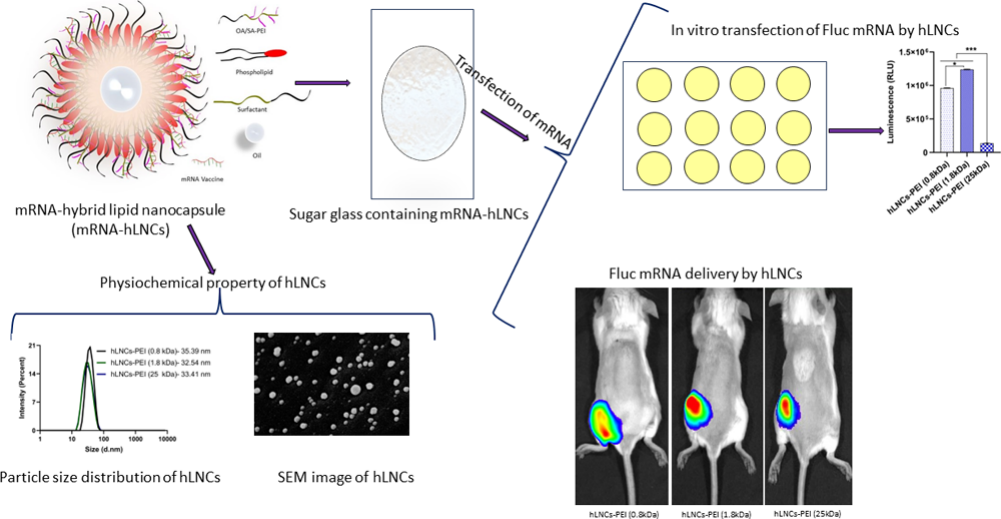

The success of the mRNA vaccine against COVID-19 has garnered significant interest in the development of mRNA therapeutics against other diseases, but there remains a strong need for a stable and versatile delivery platform for these therapeutics. In this study, we report on a family of robust hybrid lipid nanocapsules (hLNCs) for the delivery of mRNA. The hLNCs are composed of kolliphore HS15, labrafac lipophile WL1349, 1,2-dioleoyl-*sn*-glycero-3-phosphoethanolamine (DOPE), and a conjugate of oleic acid (OA) and polyethylenimines of varying size (PEI—0.8, 1.8, and 25 kDa). They are prepared by a solvent-free, temperature-phase inversion method, yielding an average size of ∼40 nm and a particle distribution index (PDI) < 0.2. We demonstrate that the PDI remains <0.2 over a wide pH range and in a wide range of medium. We further show that the PDI and the functionality of mRNA condensed on the particles are robust to drying in a sugar glass and subsequent rehydration. Finally, we demonstrate that mRNA-loaded hLNCs yield reasonable transfection in vitro and in vivo settings.

## 1 Introduction

Many types of vaccines have been developed since Edward Jenner first treated smallpox with pus from cowpox blisters.^1,2^ Messenger RNA (mRNA), discovered in the early 1960s,^3^ is a recent and significant addition to the vaccine toolbox. Owing to the fact that mRNA vaccines are amenable to rapid development and scaleup, and have good efficacy and safety profiles.^4^ mRNA vaccines against COVID-19 have shown its potential. Moreover, mRNA vaccines against influenza, HIV, tuberculosis, cancer, food, and environmental allergies are already in clinical trials or ready for clinical application^5^ after only a couple of years of development.

Most of these new vaccines use lipid nanoparticles (LNPs) as a delivery technology. LNPs have emerged as a promising mRNA delivery system because of several advantages, including protection of mRNA from degradation, efficient cellular uptake, and facilitated intracellular release.^6^ LNPs typically comprise five main components: ionizable lipids, cholesterol, a polyethylene glycol (PEG) lipid, a helper lipid, and the mRNA payload. The ionizable lipid plays a critical role in encapsulating mRNA, and endosomal escape for efficient cytosolic mRNA delivery.^7,8^

Despite incorporation in FDA-approved vaccines, ionizable lipids face several challenges, such as acute immune response, long-term toxicity, and laborious synthesis.^9^ Furthermore, while LNPs have shown great promise for mRNA delivery, there are some limitations associated with their use, such as their limited capacity for mRNA payloads, which can restrict the delivery of larger mRNA sequences or multiple mRNA constructs. The chosen lipid composition can affect both the encapsulation efficiency and the overall payload capacity of LNPs. Furthermore, functionalizing the LNPs for targeting specific tissues or cell is challenging.^6,10^ While LNPs can passively accumulate in specific tissues, achieving precise targeting may require additional modifications or ligands on the LNP surface, which may not be easily feasible. Moreover, mRNA vaccines are highly labile,^11,12^ and a major limitation of mRNA/LNPs is the need for ultracold storage conditions during storage to retain efficacy and acceptable shelf life.^6^ Therefore, there is an urgent need for an alternative nanoplatform that is free of the above-mentioned limitations but has a similar or higher potential for mRNA delivery.

In addition to LNPs, mainly cationic lipid or polymer nanoparticles have been explored for the delivery of nucleic acid. The ability of cationic lipids such as *N*-[1-(2,3-dioleyloxy)propyl]-*N*,*N*,*N*-trimethylammonium chloride (DOTMA), *N*-[1-(2,3-dioleoyloxy)propyl]-*N*,*N*,*N*-trimethylammonium chloride (DOTAP) and cationic polymers such as polyethylenimines (PEIs), poly-l-lysine, protamine to condense nucleic acid have made them exploratory targets for the delivery of mRNA. Among these, PEIs have been extensively investigated due to their high nucleic acid condensation, pH buffering capacity, and excellent transfection efficiency.^13−15^ The unique properties of PEIs/PEI nanoparticles also facilitate endosomal escape, which is essential to achieve high gene transfection. Despite these advantages, PEIs have not gained much interest because they are nondegradable and have a potential for toxicity that increases with their molecular weight.^16^ To reduce the toxicity, researchers designed degradable PEIs by covalently linking them with biodegradable moieties such as fatty acids for intracellular degradation such as hydrolysis, low endosomal pH-dependent hydrolysis, enzymatic degradation, and cytosolic reductive action by glutathione.^17^

In this study, we present a robust and biodegradable mRNA delivery nanoplatform based on engineered hybrid lipid nanocapsules (hLNCs), a hybrid structure of lipids and polymer. hLNCs present a compelling strategy in nucleic acid delivery systems by leveraging the advantages of both lipids and polymer nanoparticles. Their unique composition offers improved stability compared to single-component systems, as lipids provide structural stability, while polymers contribute additional robustness. This hybrid approach allows for better control over nucleic acid (mRNA) loading and release kinetics. The biocompatible nature of lipids combined with the tailored biocompatibility of specific polymers ensures a safer profile for medical applications. The ability to engineer these nanocapsules with targeting ligands may facilitate precise delivery of nucleic acid (mRNA) to specific tissues or cells. Moreover, fine-tuning physicochemical properties such as size, surface charge, and morphology enables control over factors such as circulation time and biodistribution. hence, the synergistic integration of lipids and polymers in hybrid nanocapsules provides a multifaceted approach to address challenges in mRNA vaccine delivery, offering enhanced stability, controlled release, versatility, and improved biocompatibility for therapeutic purposes. The hLNCs incorporate reduced-toxicity PEIs that are conjugated with oleic acid via an amide bond. The hLNCs are composed of kolliphore HS15, labrafac lipophile WL1349, 1,2-dioleoyl-*sn*-glycero-3-phosphoethanolamine (DOPE), a complex of oleic acid (OA) and different molecular weights of polyethylenimines (PEI—0.8 1.8, and 25 kDa). The fabricated hLNCs were characterized for physicochemical properties, mRNA condensation capacity, in vitro transfection in different cell lines, and in vivo transfection in mice. Furthermore, we demonstrate their robust stability in different sugar glasses at room temperature.

## 2 Material and Methods

### 2.1 Materials

Labrafac lipophile WL-1349 and 1,2-dioleoyl-*sn*-glycero-3-phosphoethanolamine (DOPE) were kindly gifted from Gattefosse Germany and Lipoid Germany, respectively. Kolliphore HS15, polyethylenimine (PEI) (average *M*_w_ ∼ 0.8 and 25 kDa), Oleic acid, Dulbecco’s modified Eagle’s medium (DMEM), Nile red, penicillin/streptomycin solution, 3-(4,5-dimethyl-2-thiazolyl)-2,5-diphenyl-2*H*-tetrazolium bromide (MTT), trypsin-EDTA (1×), poly(vinyl alcohol) (PVA), tricine, trehalose, sucrose, stachyose, and glycerol were purchased from Sigma-Aldrich (St. Louis, MO, USA). Polyethylenimine (PEI) (average *M*_w_ ∼ 1.8 kDa) was purchased from Alfa Aesar, USA. *N*,*N*′-dicyclohexylcarbodiimide (DCC), and *N*-hydroxysuccinimide (NHS) were purchased from Thermo Fisher Scientific (Suwanee, GA, USA). Ethidium bromide (EtBr), Hoechst 33342, and H_2_DCFDA were purchased from Invitrogen (Thermo Fisher Scientific, Suwanee, GA, USA), USA. Agarose molecular biology grade was purchased from IBI Scientific. Firefly Luciferase (FLuc) mRNA was purchased from TriLink Biotechnologies (San Diego, California, USA). Other chemicals and solvents used in the work were analytical grade and were used as procured.

### 2.2 Methods

#### 2.2.1 Synthesis of the Oleic Acid-PEI Conjugate

The synthesis of oleic acid-PEI conjugate was conducted according to established procedures with minor adjustments.^18^ Initially, a reaction mixture comprising oleic acid and the coupling agent *N*,*N*′-dicyclohexylcarbodiimide (DCC) dissolved in anhydrous dichloromethane (DCM) was prepared. To this mixture was added an equimolar quantity of *N*-hydroxysuccinimide (NHS) and the reaction proceeded under an inert nitrogen atmosphere at room temperature for an overnight duration. Following this, the resultant dicyclohexylurea byproduct was removed via filtration, and the filtrate containing activated oleic acid was collected. Subsequently, a coupling reaction with polyethylenimines (PEIs) of molecular weights of 0.8, 1.8, and 25 kDa was performed in the presence of triethylamine at room temperature over another overnight period. Upon completion, the oleic acid-PEI conjugate was precipitated by the addition of an excess quantity of diethyl ether. The resulting precipitate was washed with diethyl ether to remove impurities, and the purified oleic acid-PEI conjugate was subsequently dried under a vacuum and stored at 4 °C for further utilization.

#### 2.2.2 Preparation of Sugar Glass

Sugar glass was prepared by dissolving sugar (20%, w/v), poly(vinyl alcohol)/tricine (5.0%, w/v), and glycerol (5.0%, w/w of sugar) in ultrapure water. The sugar glass composition detail is presented in Table S1. Subsequently, hLNCs and mRNA were dispersed. 50 μL aliquots of sugar solution with bioactive components were dried on coverslips under a hood at room temperature for 24 h, at which time they were transferred to a desiccator for complete drying and stored under desiccation until use.

#### 2.2.3 Engineering Hybrid Lipid Nanocapsules (hLNCs) and Preparation of the mRNA-hLNCs Complex

hLNCs were developed by following previously reported methods with slight modifications.^19,20^ Briefly, Labrafac lipophile WL1349, kolliphore HS 15, DOPE, and OA-PEI conjugate (oleic acid conjugate with different molecular weights of PEI) were mixed in 4:4:1:1 mass ratio, respectively. Subsequently, saline (20% w/v, 1 mL/g of hLNCs preparation) was added, and the mixture was subjected to a three heating and cooling cycle (50 ↔ 70 °C) under a magnetic stirring hot plate, making sure in each cycle to achieve the phase inversion temperature. At the end of the third cycle, cold water was added and left for 5 min under magnetic stirring to prepare hLNCs. The obtained hLNCs were purified by dialysis (MWCO-100 kDa, Spectrum G235059) for 48 h with an intermittent water change, and the purified hLNCs were stored at 4 °C.

To prepare the mRNA-hLNCs complex, a specific amount of hLNCs was dispersed in a sugar solution followed by the addition of a suitable amount of mRNA. After gentle pipetting, it was allowed to make a complex for 30–45 min at room temperature, followed by drying to form a sugar glass as described above.

#### 2.2.4 Particle Size and Zeta Potential

Particle size and zeta potential of hLNCs were measured by Zetasizer Nano ZS (Malvern Instrument).^21^ A suitable concentration (1–5 mg/mL) of hLNCs was prepared in ultrapure water in triplicate and analyzed.

#### 2.2.5 Cryo-Scanning Electron Microscopy (Cryo-SEM)

The hLNC morphology was evaluated by Cryo-SEM (Thermo Fisher Helios 5CX, FIB-SEM) as reported previously.^22^ The diluted hLNCs sample was drop-cast on the stub, frozen in a liquid nitrogen chamber, sublimed, and sputter-coated with platinum. SEM images of the samples were captured at 5 kV.

#### 2.2.6 Ethidium Bromide-Nucleic Acid Exclusion Assay

EtBr intercalation with mRNA was determined by a multimode plate reader (BioTek) at λ_ex_/λ_em_= 510/590 nm.^23^ Briefly, different known concentrations of free mRNA (0.025, 0.050, 0.10, 0.20, 0.4, 0.8, 1.6, and 3.2 μg/mL) were incubated with EtBr. The mean fluorescence intensity was determined, data were fitted into linear regression, and the first-order equation (standard plot) was generated using GraphPad Prism. Similarly, to determine the concentration of mRNA in an unknown sample, EtBr was incubated with mRNA, fluorescence was measured, and the concentration was evaluated with the standard plot.

#### 2.2.7 Agarose Gel Electrophoresis

Agarose gel electrophoresis was performed by using 1.0% (w/v) agarose gel. Briefly, agarose gel was prepared in Tris base, acetic acid, and EDTA (TAE) buffer containing 0.5 μg/mL of EtBr. Free mRNA and/or mRNA-hLNCs complex was loaded into wells along with loading dye (Bromophenol blue, 6X). It was electrophoresed using an electrophoresis unit (Bio-Rad, USA) at 80 V for 15–20 min. The mRNA was tracked using a ChemiDoc Imaging System (Bio-Rad)

#### 2.2.8 mRNA Condensation Capacity of hLNCs

The mRNA condensation capacity of hLNCs was determined by an ethidium bromide-nucleic acid exclusion assay and visualized by a gel electrophoresis assay. Briefly, mRNA-hLNCs complexes with different ratios of mRNA and hLNCs (hLNCs-PEI-0.8 kDa; 1:10, 1:20, 1:30, 1:40. hLNCs-PEI-1.8 kDa; 1:5, 1:10, 1:20, 1:30. hLNCs-PEI-25 kDa; 1:2.5, 1:5, 1:10, 1:20) were prepared as described previously. Furthermore, free mRNA was quantified by an ethidium bromide-nucleic acid exclusion assay. The percentage condensation of mRNA was calculated using the following equation.
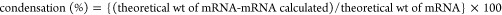


To visualize the uncondensed mRNA, agarose gel electrophoresis was performed as described previously.

#### 2.2.9 Robustness of hLNCs and mRNA Condensed with hLNCs to Drying in Sugar Glasses

Sugar glasses containing hLNCs or mRNA-hLNCs were prepared as described above. To extract the hLNCs/mRNA from the sugar glass, the glass was dissolved in ultrapure water. The stability of the hLNCs was determined by the retention of their initial size distribution. The stability of mRNA was tested by agarose gel electrophoresis. After decomplexing mRNA from hLNCs using SDS (0.5% w/v), mRNA was subsequently subjected to agarose gel electrophoresis. mRNA bands were visualized, and their optical density was analyzed using ImageJ (an open software).

#### 2.2.10 Cell Culture

The different cell lines HaCaT, Raw 264.7 (high glucose DMEM), and DU145 (RPMI) were cultured in vitro. The medium was supplemented with 10% (v/v) FBS and 1% antibiotics. They were grown in a humidified incubator supplemented with 5% CO_2_. For further experiments, following standard protocols, cells were harvested using trypsin-EDTA/or scrapping (Raw 264.7) and seeded in respective cell culture plates.

#### 2.2.11 MTT Assay

In vitro, the cytotoxicity of hLNCs was determined using the MTT assay in three cell lines mentioned above by following the previously reported method.^24,25^ They were seeded at a density of 5000 cells/well in standard 96-well cell culture plates and allowed to attach overnight. Cells were treated with different concentrations of hLNCs and incubated for 24 h. After that, the medium was removed and cells were treated with serum-free medium containing MTT reagent (0.5 mg/mL). After 3 h, the medium was removed carefully, formed formazan crystals were dissolved in 100 μL of dimethyl sulfoxide (DMSO), absorbance was recorded at 570 nm, and % cell viability was calculated.

#### 2.2.12 Reactive Oxygen Species (ROS) Assay

DU145, HaCaT, and Raw264.7 cells were seeded in 12 well plates at a confluency of 70–80% and they were allowed to attach overnight. Cells were treated with different concentrations of hLNCs (50, 100, and 150 μg/mL) for 1 h and 24 h. After that, hLNCs were replaced with H_2_DCFDA, and the cells were incubated for another 45 min. Subsequently, cells were harvested followed by washing with PBS (1×, pH-7.4) and analyzed by a flow cytometer (BD, Fortessa). Cells without treatment and treatment with H_2_O_2_ were considered as negative and positive control, respectively.

#### 2.2.13 In Vitro Cellular Uptake

In vitro cellular uptake of hLNCs was evaluated in the cell lines mentioned previously using flow cytometry and confocal microscopy. Briefly, cells at a density of 1 × 10^5^ cells/well in a 12-well tissue culture plate were seeded and allowed to attach overnight. The cells were treated with hLNCs loaded with Nile Red for 1, 3, 6, and 24 h. They were washed with cold PBS (1×, pH-7.4) and harvested using trypsin-EDTA/or scrapping. After the harvested cells were washed, they were analyzed by flow cytometry. The fluorescent intensity of 10,000 cells was recorded, and the mean fluorescence was calculated.

Additionally, cells were imaged by using confocal microscopy (ZEISS, LSM 900) to visualize the internalization. Briefly, cells were seeded on treated coverslip and allowed to grow for 24 h. The cells were treated with hLNCs loaded with Nile Red for 3 h. Subsequently, cells were washed with cold PBS followed by nuclear counter stain and fixation using Hoechst 33342 and formaldehyde solution (4% w/v), respectively, and fluorescent images were captured.

#### 2.2.14 In Vitro Firefly Luciferase Transfection Assay

The transfection of FLuc mRNA-hLNCs was evaluated in the three cell lines mentioned above. Briefly, all cells with suitable density (80–90% confluency) were seeded in 12 well plates and allowed to attach overnight. The cells were treated with mRNA-hLNCs (equivalent to 1 μg/well of mRNA) for 24 h. Lipid nanoparticles loaded with mRNA (mRNA-LNPs) and free mRNA were kept as positive and negative controls, respectively. After that, the cells were washed with PBS and lysed. The expressed luciferase was determined by Luciferase Reporter Gene Detection Kit (Luc1, Sigma-Aldrich) following the manufacturer’s protocol using a multimode plate reader. The luminescence signal was normalized per milligram of protein (cell lysate).

#### 2.2.15 In Vivo Firefly Luciferase Expression Assay

The expression study was performed in female Balb/c mice according to a protocol approved by the IACUC, Georgia Institute of Technology. 6–8-Weeks-old mice were intramuscularly (IM) administered with mRNA-hLNC suspension (equivalent to 1.5 μg of mRNA) in saline (0.9% w/v). mRNA-LNPs and free mRNA (equivalent to 1.5 μg of mRNA) were kept as positive and negative controls, respectively. After 24 h, 100 μL of a luciferin D solution (15 mg/mL) was injected intraperitoneally (IP). Subsequently, bioluminescence imaging was carried out with an IVIS Spectrum CT Imaging System (PerkinElmer).

### 2.3 Statistical Analysis

Results are reported as mean ± standard deviation (SD). The obtained results were analyzed by performing one-way ANOVA or student’s *t* test. The level of confidence was kept at 95%, *p*-value <0.05 was considered as statistically significant (*p< 0.05,**p< 0.01, and***p< 0.001).

## 3 Results and Discussion

### 3.1 Synthesis and Characterization of the OA-PEI Conjugate

The conjugate of OA-PEI was synthesized using carbodiimide chemistry, a reaction between PEI (*M*_w_ ∼ 0.8, 1.8, and 25 kDa) and oleic acid. The ^1^H NMR spectra were recorded to investigate whether OA and PEI were successfully conjugated together and obtained data was represented in supplementary figures. The ^1^H NMR spectrum of OA showed several peaks around 0.865, 1.312, 1.493, 2.195, 1.497, and 5.33 ppm, which correspond to the proton of the OA skeleton −CH_3_, CH_2_–CH_2_–, CH=CH, etc., respectively (Supplementary Figure S1a). The characteristic peak of −COOH of OA showed at 11.958 ppm.^26^ The ^1^H NMR spectra of PEI (*M*_w_ 0.8, 1.8, and 25 kDa) detected around 0.8–1.0 ppm (−CH_3_), 1.0–1.3 ppm (CH_3_(−CH_2_)_5_−), (−CH_2_CH_2_CH=CH−) and (−CH=CH–CH_2_–(CH_2_)_3_−), 1.7–1.9 ppm (−CH_2_CH_2_CO−), 2.3- 2.4 (−CH_2_CH=CHCH_2_−), 5.34 (−CH=CH−) (Supplementary Figures S1b and S2a,b). In the OA-PEI conjugate, the peaks from 0.85 to 5.33 ppm included the proton of the OA and PEI backbone. The new peak at 3.388 ppm (OA-PEI-0.8 kDa), 3.710 ppm (OA-PEI-1.8 kDa), and 3.362 ppm (OA-PEI-25 kDa) indicated that the amino group of PEI and the carboxyl group of oleic acid were reacted to form OA-PEI conjugate (Supplementary Figures S3a,b and S4).^27^ The degree of modification of PEI was determined by integrating the peaks corresponding to amine groups which revealed that the degree of modification is 23.82, 10.11 and 3.41% in OA-PEI (0.8), OA-PEI (1.8), and OA-PEI (25 kDA) conjugates, respectively. We varied the degree of modification of polyethylenimine (PEI) with oleic acid based on the molecular weight of PEI. Higher molecular weight PEI (25 kDa) required less oleic acid modification compared to lower molecular weight PEI (0.8 kDa) to achieve optimal handling during preparation and optimal characteristics of the finished nanocapsules. This adjustment is necessary to balance the surface charge, hydrophobicity, and size of hLNCs, influencing their stability and performance in various mRNA delivery cases.

### 3.2 Preparation and Characterization of hLNCs

**Figure 1:**
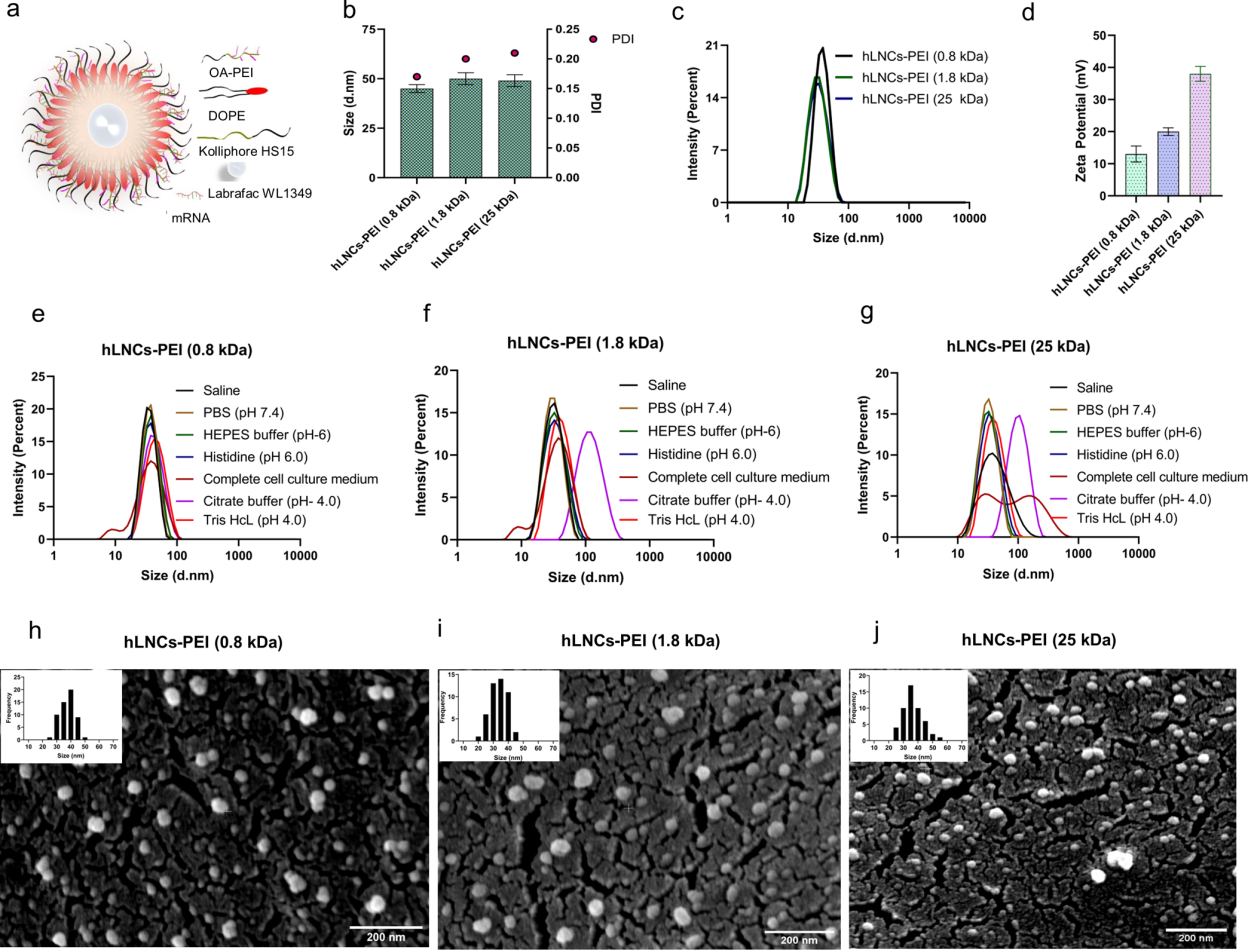
(a) Schematic of hLNCs. (b) Average size and PDI of hLNCs engineered with OA-PEI (*M*_w_∼ 0.8, 1.8, and 25 kDa) (*n* = 3 independent development). (c) Particle distribution of hLNCs. (d) Zeta potential of hLNCs measured in ultrapure water. (e–g) Particle distribution of hLNCs-PEI (0.8), hLNCs-PEI (1.8), and hLNCs-PEI (25 kDa), respectively, in different media. (h, i, and j) Cryo-SEM micrograph of hLNCs-PEI (0.8), hLNCs-PEI (1.8), and hLNCs-PEI (25 kDa), respectively. The scale bar of the Cryo-SEM micrograph represents a distance of 200 nm.

hLNCs were prepared by a solvent-free PIT method, a technique used for the preparation of emulsions or emulsion-based formulations. It involves inducing a phase transition or inversion of the emulsion by manipulating the temperature. In the preparation of hLNCs, saline was used as the dispersed phase, and kolliphore HS-15, DOPE, labrafac lipophile WL1349, and OA-PEI (an oleic acid conjugate of different molecular weight of PEI; ∼ 0.8, 1.8, and 25 kDa) were used as a continuous phase which together forms a robust hLNCs platform by applying the PIT method. Figure. 1a represents the schematic of the hLNCs, a hybrid structure between polymeric nanocapsules and liposomes. As shown in Figure. 1a, the core of hLNCs consists of labrafac lipophile WL1349 and other ingredients (kolliphore HS15, DOPE, OA-PEI) to form a shell. Since it is not possible to visualize the core–shell structure directly with methods such as SEM, the core–shell structure of hLNCs was deduced through ingredient interactions determined by differential scanning calorimetry (DSC) as previously done for a similar structure.^28,29^ DSC analysis revealed endothermic peaks for DOPE at −25.19, 53.32, and 64.06 °C, Kolliphore HS15 at 23.33 °C, Labrafac lipophile WL1349 at −3.32 °C. The endothermic peak of OA-PEI-0.8 kDa, OA-PEI-1.8 kDa, and OA-PEI-25 kDa was revealed at −32.41/18.09, −21.85/55.96, and −19.44/70.65 °C, respectively (Supplementary Figure S5). In the DSC spectra of hLNCs, an endothermic peak at −4.12 represents a hydrophobic core which is largely composed of labrafac lipophile WL1349. However, a slight shift in peak might be due to the hydrophobic interaction of other ingredients in the core. Moreover, a characteristic peak, representing shell in all hLNCs was observed near 27 °C which is a merged peak of kolliphore HS15, DOPE, and OA-PEI conjugate (Figure S5g–i). This might be due to the ionic interaction of hydrophilic parts of all ingredients. Therefore, two distinguished peaks, i.e., hydrophobic and hydrophilic components, confirm a core–shell structure of hLNCs. Moreover, the DSC spectra of the physical mixture of ingredients showed distinguish peaks of all ingredients with slight shifting in the DOPE peak (Supplementary Figure S6g–i). This further confirms that the shell of hLNCs is composed of strong ionic interaction of DOPE, PEI, and kolliphore HS15 while, in the physical mixture, it is absent.

Unlike liposomes, hLNCs are highly stable because of their hybrid structure. The basic structure of the components of hLNC’s shell is similar to that of liposomes in that they have a hydrophobic tail (fatty acid) and a hydrophilic head. While engineering hLNCs, the hydrophobic tail of all ingredients entangled in the triglyceride forms the core and the hydrophilic head forms the shell of hLNCs, thereby providing a robust structure. The kolliphore HS-15, a nonionic surfactant, is composed of fatty acid and PEG molecules (polyethylene glycol (15) 12-hydroxy stearate).^30^ The fatty acid component of it participates in the development of the core, and PEG molecules orient on the surface, providing dispersion stability. PEIs are well-proven for the delivery of nucleic acid,^14,31^ and we have employed OA-PEI conjugates in hNLCs, which are mainly responsible for the loading/condensing of mRNA. Keeping all the ingredient amounts the same, but different molecular weight PEI and oleic acid conjugate (OA-PEI; 0.8, 1.8, and 25 kDa), hLNCs were prepared and their size/zeta potentials were measured. There was no significant effect of different OA-PEI conjugates on the average capsule size and particle distribution (Figure 1b,c). Unlike size, there were significant differences in the zeta potential of hLNCs-PEI (0.8), hLNCs-PEI (1.8), and hLNCs-PEI (25 kDa), which was found to be 13 ± 2, 20 ± 3, and 38 ± 5 mV, respectively (Figure 1d). Increasing the molecular weight of PEI increases the number of amine groups, thereby increasing the positive charge density on the capsules.

To assess formulation stability under different conditions, the size distribution of hLNCs was measured in different mediums under a range of pH values (pH 4 to 7.4); the data is presented in Figure 1e–g. No significant change in the size or distribution of hLNCs was observed except in citrate buffer at pH 4.0. The entire distribution of hLNCs-PEI (1.8 and 25 kDa) sizes shifted to higher values without disruption of the particles (PDI < 0.3) (Figure 1f, g). This could be due to the charge interaction between citrate and PEI (1.8 and 25 kDa) which is not significant with the lower molecular weight of PEI (0.8 kDa).

To confirm the structural stability and the potential to withstand the drying stress, hLNCs were dispersed in a sugar glass solution, and sugar glasses were prepared by drying at room temperature, as described above. hLNC-bearing sugar glasses were dissolved, and capsule size was measured. No significant changes in average capsule size were observed (Supplementary Figure S7a–c), indicating the robust structure of the hLNCs. The morphology and particle size of the hLNCs-PEI (0.8, 1.8, and 25 kDa) were assessed using cryo-SEM, revealing spherical and homogeneous distributed particles (Figure 1h–j). The measured particle distribution (inset figure of SEM images) is closely correlated with those determined by DLS (Figure 1c), although with a relatively lower average particle size compared to the average size measured by DLS. However, slight discrepancies in particle sizes measured by SEM and DLS are anticipated, given the utilization of distinct measurement techniques.^28,32^

### 3.3 mRNA Condensation Capacity and Stability of mRNA in Sugar Glasses

**Figure 2:**
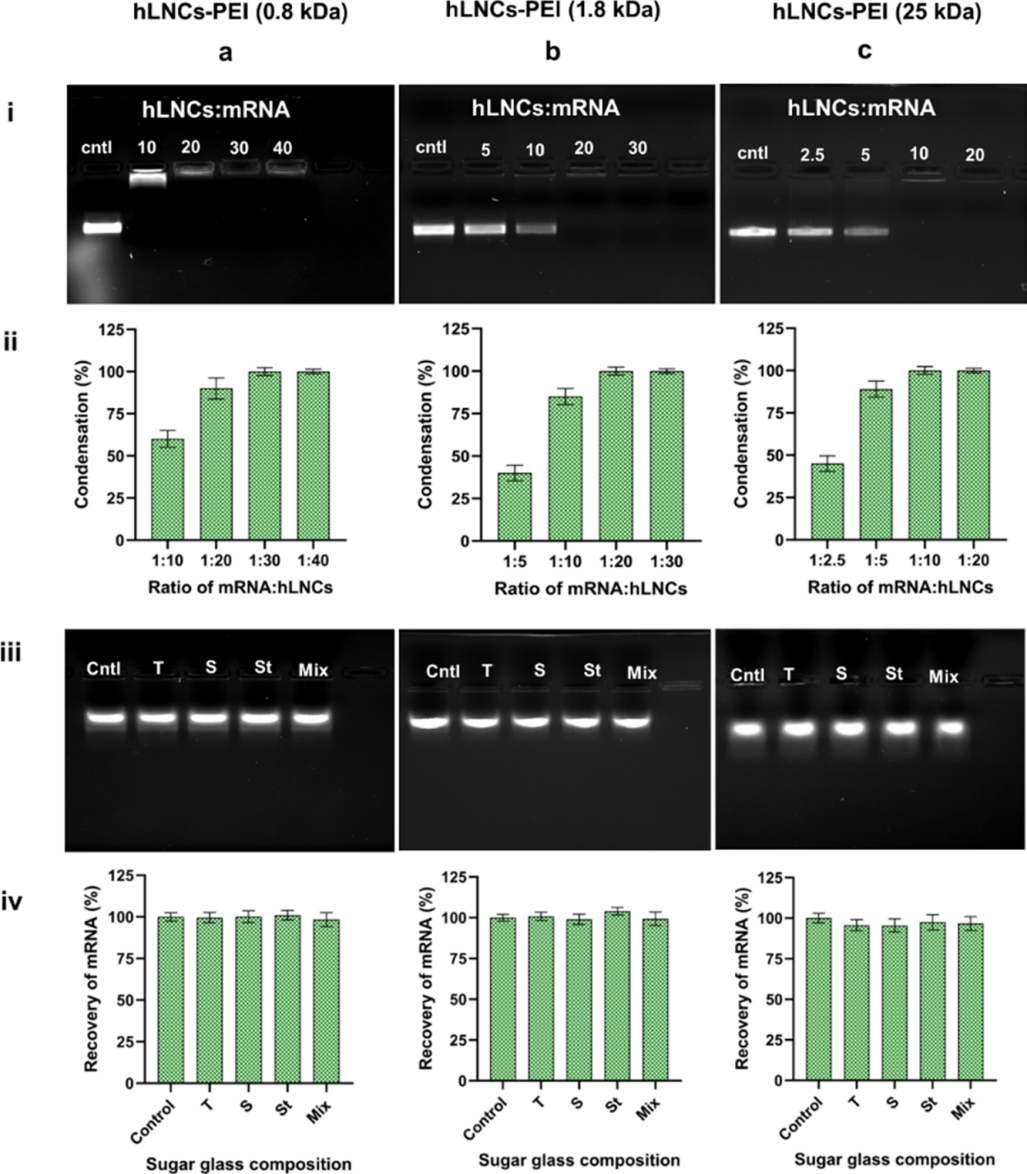
(i) mRNA condensation capacity of hLNCs visualized by agarose gel electrophoresis, (ia, ib, and ic) condensation capacity of hLNCs-PEI (0.8), hLNCs-PEI (1.8), hLNCs-PEI (25 kDa), respectively. (ii) mRNA condensation capacity hLNCs determined by EtBr exclusion assay, (iia, iib, and iic) condensation capacity of hLNCs-PEI (0.8), hLNCs-PEI (1.8), hLNCs-PEI (25 kDa), respectively. (iii) Stability of mRNA in sugar glass by gel electrophoresis; images of mRNA-hLNCs from different sugar glasses. (iiia, iiib, and iiic) Gel electrophoresis of mRNA-hLNCs-PEI (0.8), mRNA-hLNCs-PEI (1.8), and mRNA-hLNCs-PEI (25 kDa), respectively, from different sugar glasses. T, S, St, and mix stand for trehalose, sucrose, stachyose, and a mixture of these sugars. (iv) Percent mRNA recovery analyzed by ImageJ, the optical density of gel electrophoresis of mRNA-hLNCs from different sugar glasses. (iva, ivb, and ivc) Recovery of mRNA from hLNCs-PEI (0.8), hLNCs-PEI (1.8), and hLNCs-PEI (25 kDa), respectively.

mRNA condensation is one of the critical aspects of delivering mRNA-based therapeutics and vaccines efficiently into target cells. Condensing mRNA into nanoparticles helps protect the fragile mRNA from degradation by enzymes and other factors present in the extracellular environment, which increases its stability and shelf life.^33^ The hLNCs, engineered with different molecular weights of PEI, were evaluated for their mRNA condensation capacity by gel electrophoresis and the EtBr exclusion assay. Around 1:30, 1:20, and 1:10 ratio of mRNA to hLNCs-PEI (0.8), hLNCs-PEI (1.8), and hLNCs-PEI (25 kDa), respectively, was required to complete condensation of mRNA (Figure 2i, ii). Additionally, the zeta potential of the complexes prepared with different ratios of mRNA to hLNCs was measured and the data is presented in supplementary Figure S8. mRNA-hLNCs complexes prepared with excess mRNA showed negative zeta potential, while complexes prepared with an excess amount of hLNCs showed positive zeta potential (Supplementary Figure S8a–c). In the ratio where 100% mRNA condensation was observed by gel electrophoresis or EtBr exclusion assay, zeta potentials were found close to zero which complements the results obtained from gel electrophoresis (Figure 2i) and EtBr exclusion assay (Figure 2ii). In agreement with this, the condensation capacity of hLNCs showed the following ordering: hLNCs-PEI (25) > hLNCs-PEI (1.8) > hLNCs-PEI (0.8 kDa). The higher condensation capacity of hLNCs composed of a higher molecular weight of PEI might be due to the higher molecular weight of PEI which contains a higher free amine group and gives a higher positive charge density which facilitates the condensation of a higher amount of mRNA.

The results presented above confirm that engineered hLNCs are robust and maintain their structure, even after they are incorporated into sugar glasses. Taking the next step, hLNCs containing mRNA (mRNA-hLNCs) were incorporated into sugar glasses, and recovery of intact mRNA was evaluated using gel electrophoresis by dissolving the glasses followed by decomplexing mRNA from hLNCs. From Figure 2iii/iv(a–c), it can be concluded that more than 98% of the loaded mRNA was intact after drying in the sugar glass. This result confirms that mRNA-hLNCs may be stored in a sugar glass. It has been shown that storing biomolecules in sugar glass can significantly increase their shelf-life, even at room temperature or higher.^34,35^

### 3.4 Cellular Uptake Kinetics

**Figure 3:**
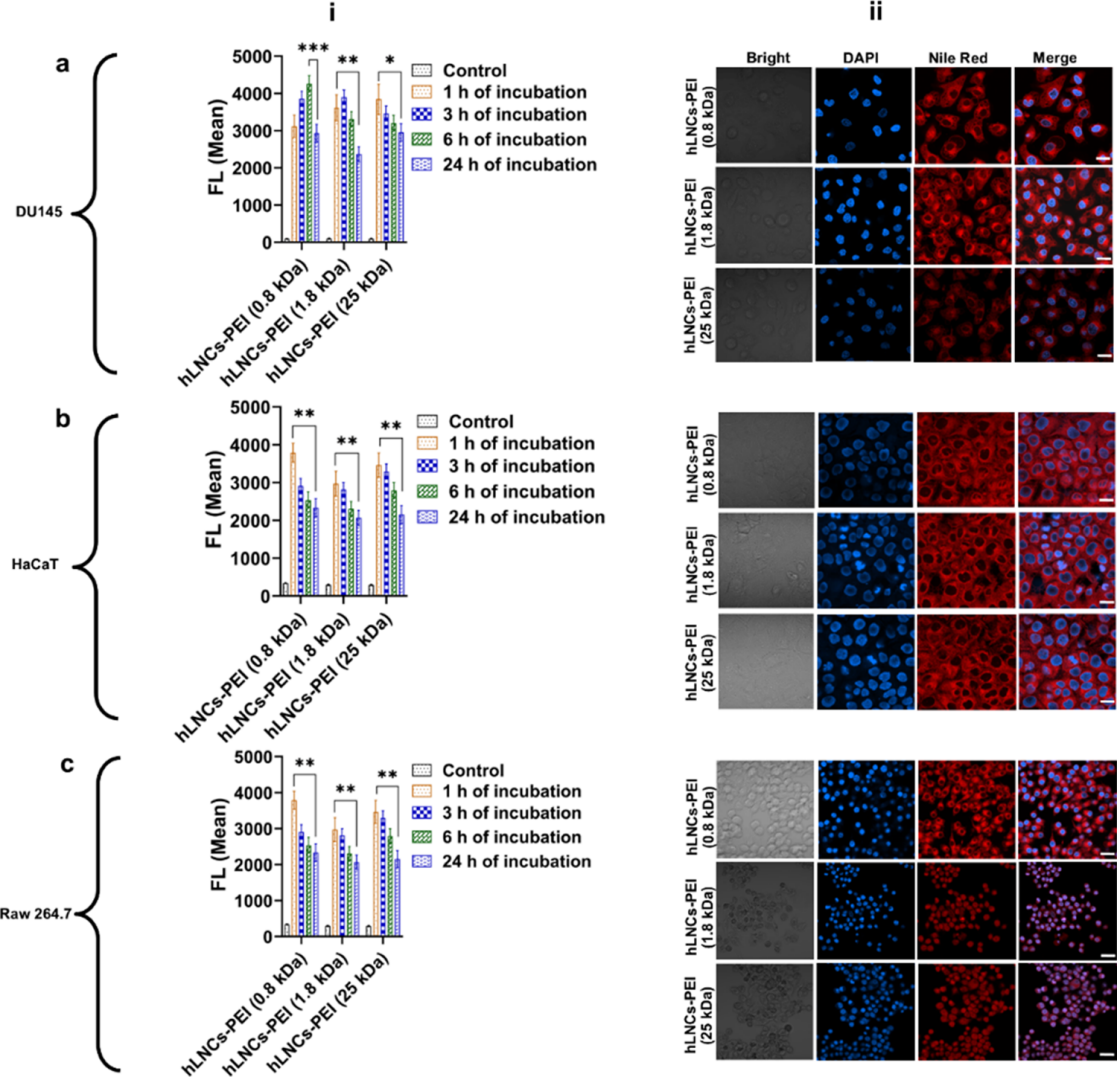
Cellular uptake of hLNCs in different cell types. (a), (b), and (c) represent cellular uptake in DU145, HaCaT, and Raw264.7 cells, respectively, measured by flow cytometry and confocal microscopy. (ai, bi, and ci) Cellular uptake kinetics of hLNCs in DU145, HaCaT, and Raw264.7 cells, respectively, incubating for 1, 3, 6, and 24 h by flow cytometer. (aii, bii, and cii) Cellular uptake of hLNCs, confocal micrograph of DU145, HaCaT, and Raw264.7 cell, respectively. The scale bar in the confocal images represents a size of 20 μm.

Cellular uptake refers to the process by which substances are internalized by cells and transported across the cell membrane into the intracellular environment. mRNA needs to act within the cells to exert its effects as mRNA-based therapies rely on the cells’ machinery to produce the desired therapeutic protein.^36^ Hence, cellular uptake ensures that the therapeutic agent is delivered to the appropriate cellular compartment for proper action. Therefore, before performing the transfection efficiency of mRNA-hLNCs, we evaluated the cellular uptake kinetics of the hLNCs in different cell types (DU145, HaCaT, and Raw 264.7) using flow cytometry and confocal microscopy. The obtained result is represented in Figure. 3. The cellular uptake kinetic pattern of hLNCs-PEI (0.8 kDa), hLNCs-PEI (1.8 kDa), and hLNCs-PEI (25 kDa) is similar in HaCaT and Raw264.7 cells. A maximum uptake occurred in the initial 1 h followed by significantly declined cellular uptake (Figure 3bi, ci). However, in a cancer cell line (DU145), the uptake of hLNCs-PEI (0.8 kDa) and hLNCs-PEI (1.8 kDa) achieved a peak in 6 and 3 h, respectively (Figure 3ai). Moreover, hLNCs-PEI (25 kDa) showed a similar pattern to other cells as it achieved a peak in 1 h (Figure 3ai). The phenomena of rapid cellular uptake followed by subsequent decline over time might be attributed to several factors such as a combination of efficient initial adsorption, potential saturation of endocytic pathways, cellular response dynamics, and intracellular processing highlighting the dynamic and complex interplay between hLNCs and cellular systems.^37^

Furthermore, confocal microscopy was performed to visualize the uptake of Nile-Red loaded hLNCs. It can be confirmed from Figure 3aii, bii, cii that hLNCs are taken up well by all cell types, further strengthening the results obtained from flow cytometry. While comparing the maximum uptake between hLNCs-PEI (0.8), hLNCs-PEI (1.8), and hLNCs-PEI (25 kDa), it was observed that all cell types showed significantly higher uptake of hLNCs-PEI (0.8 kDa) compared to others. This might be due to the intermediate-level zeta potential of hLNCs-PEI (0.8 kDa). Either the very high or very low zeta potential of nanoparticles may not be promising for adequate cellular uptake.^38^ The hLNCs-PEI (1.8 kDa) and hLNCs-PEI (25 kDa) have higher zeta potentials than hLNCs-PEI (0.8 kDa).

### 3.5 In Vitro Cellular Toxicity

**Figure 4:**
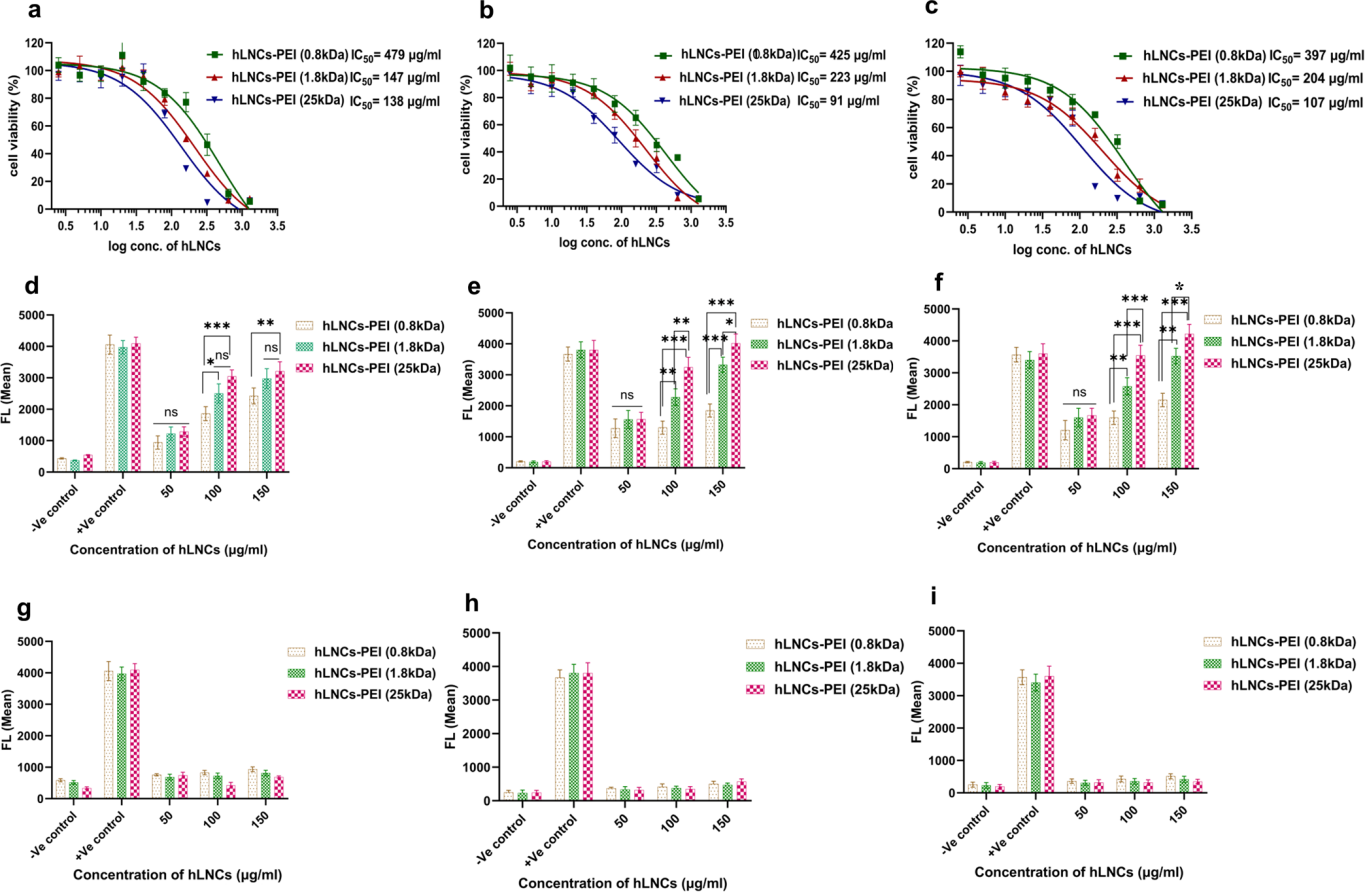
Cellular toxicity and ROS production of hLNCs in DU145, HaCaT, and Raw264.7 cells. (a, b, and c) Toxicity of hLNCs-PEI (0.8), hLNCs-PEI (1.8), and hLNCs-PEI (25 kDa) in DU145, HaCaT, and Raw264.7 cells, respectively. (d, e, and f) Relative ROS production of different concentrations of hLNCs-PEI (0.8), hLNCs-PEI (1.8), and hLNCs-PEI (25 kDa) in DU145, HaCaT, and Raw264.7 cells, respectively, after 1 h of incubation. (g, h, and i) Relative ROS production of different concentrations of hLNCs-PEI (0.8), hLNCs-PEI (1.8), and hLNCs-PEI (25 kDa) in DU145, HaCaT, and Raw264.7 cells, respectively, after 24 h of incubation.

Nanoparticles with toxic properties could induce undesirable side effects or damage healthy cells, and it is essential to evaluate hLNCs biocompatibility and potential toxicity in cells. Cellular toxicity of engineered hLNCs was evaluated in different cell types; DU145, Raw264, and HaCaT by treating them with different concentrations of hLNCs for 24 h. As shown in Figure 4, all types of hLNCs showed cellular toxicity in a dose-dependent manner. The toxicity pattern of hLNCs was cell-dependent; the IC_50_ of the same hLNCs was significantly different in different cell types. hLNCs-PEI (0.8 kDa) showed less toxicity than hLNCs-PEI (1.8 kDa) and hLNCs-PEI (25 kDa) in all cell types (Figure 4a–c). This is not unexpected, as the toxicity of PEIs is known to depend strongly on their molecular weight.^17^ Our previous study confirmed that similar lipid nanocapsules induce the production of cellular reactive oxygen species (ROS) which is a major cause of toxicity in the cell.^20^ Therefore, ROS production was assessed in all mentioned cell types by treating them with different concentrations of hLNCs-PEI (0.8), hLNCs-PEI (1.8), and hLNCs-PEI (25 kDa) for 1 and 24 h. The data is shown in Figure 4.

Like toxicity, the ROS production was also concentration-dependent, significantly increasing with the concentration of hLNCs (Figure 4d–f). Moreover, we observed that the production of ROS was rapid and transient as no ROS was detected after 24 h of treatment (Figure 4g–i). This could be attributed to various reasons such as (a) hLNCs might induce a rapid burst of ROS production shortly after treatment, which then subsides over time, (b) during the first hour of treatment, a higher concentration of hLNCs was taken up and localized in cellular compartments that promote ROS production. However, over time, nanoparticles could be cleared, redistributed, or sequestered in different cellular compartments, leading to a decrease in ROS levels and (c) degradation or adaptation of hLNCs by the cells.

### 3.6 In Vitro Transfection Efficiency

As a preparatory step to in vivo transfection experiments, we performed in vitro transfection. PEIs have been demonstrated as components of effective nonviral gene transfecting vehicles, but they have drawbacks.^39^ To ameliorate these drawbacks and to incorporate the PEIs into our constructs, we modified them with oleic acid. According to previous reports, the transfection efficiency of PEIs is molecular weight-dependent, with higher molecular weight PEIs giving a higher transfection efficiency.^17^

**Figure 5:**
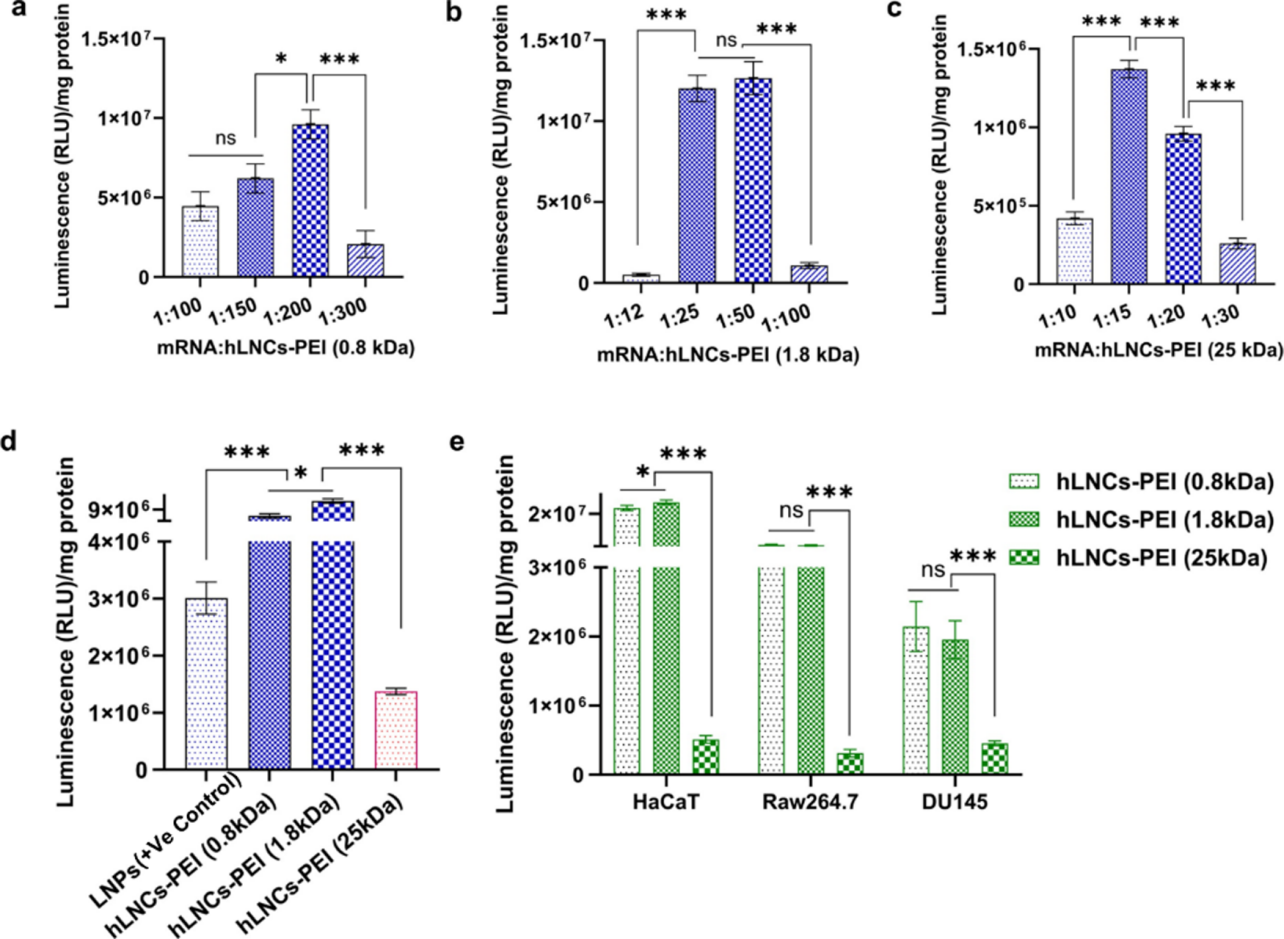
Optimization of mRNA to hLNCs ratio for optimum transfection of FLuc mRNA in HaCaT cells. (a) Transfection of mRNA using different ratio mRNA to hLNCs-PEI (0.8 kDa). (b) Transfection of mRNA using different ratios of mRNA to hLNCs-PEI (1.8 kDa). (c) Transfection of mRNA using different ratios of mRNA to hLNCs-PEI (25 kDa). (d) Comparison of the transfection efficiency of LNPs, hLNCs-PEI (0.8), hLNCs-PEI (1.8), and hLNCs-PEI (25 kDa) in HaCaT cells with the optimized ratio of mRNA to hLNCs. (e) Transfection efficiency of hLNCs-PEI (0.8), hLNCs-PEI (1.8), and hLNCs-PEI (25 kDa) in HaCaT, Raw264.7 and DU145 with optimized ratio of mRNA to hLNCs.

In this study, we first optimized the ratio of FLuc mRNA:hLNCs in HaCat cells, as the nitrogen-to-phosphate ratio (N/P) ratio affects the efficiency of complex formation between the nanoparticles and nucleic acids.^40^ This is crucial for protecting nucleic acids from degradation and facilitating cellular uptake and higher transfection efficiency. Complexes with varying ratios of FLuc mRNA and hLNCs-PEI (0.8 kDa), hLNCs-PEI (1.8 kDa), and hLNCs-PEI (25 kDa) were prepared, and their transfection efficiency was evaluated in the chosen cell types. The resulting data is represented in Figure 5. The optimal ratio of FLuc mRNA to hLNCs-PEI (0.8 kDa), hLNCs-PEI (1.8 kDa), and hLNCs-PEI (25 kDa) for in vitro transfection was observed as 1:200, 1:25/50, and 1:15, respectively (Figure 5a–c). However, with a lower or higher ratio compared to the optimal ratio, transfection efficiency was significantly decreased. Unlike PEI nanoparticles, the transfection efficiency of hLNCs engineered with lower molecular weight of PEIs (0.8 and 1.8 kDa) showed significantly higher transfection efficiency than hLNCs engineered with PEI-25 kDa (Figure 5d). Even after comparable cellular uptake of all hLNCs, transfection efficiency varied, which might be due to hLNCs composed of lower molecular weight PEI (0.8 and 1.8 kDa), forming a complex with mRNA that strikes a better balance between stability and the ability to release the cargo within cells compared to hLNCs-25 kDa. The transfection efficiency of hLNCs was also compared to LNPs (as a positive control) and significantly higher transfection efficiency of hLNCs-PEI (0.8 and 1.8 kDa) was observed compared to LNPs (Figure 5d). However, no transfection of free mRNA (the negative control) was observed. Furthermore, the transfection efficiency of hLNCs-PEI (0.8 kDa), hLNCs-PEI (1.8 kDa), and hLNCs-PEI (25 kDa) was evaluated in HaCaT, Raw264.7, and DU145 cells. Similarly, hLNCs-PEI (0.8 kDa) and hLNCs-PEI (1.8 kDa) showed significantly higher transfection efficiency compared to hLNCs-PEI (25 kDa) in other cells also (Figure 5e). The transfection efficiencies of hLNCs-PEI (0.8 kDa) and hLNCs-PEI (1.8 kDa) were not significantly different in Raw264.7 and DU145 cells, respectively. However, hLNCs-PEI (0.8 kDa) showed slightly higher transfection than hLNCs-PEI (1.8 kDa) in HaCaT cells. Rather than a similar cellular uptake behavior of hLNCs by the mentioned cells, the transfection efficiency of HaCaT cells compared to Raw264.7 or DU145 cells was observed to be higher. This might be due to unique characteristics of different cells such as intracellular trafficking mechanisms, which may influence transfection. Like other nanoplatforms, hLNCs also showed promising transfection efficiency across all cell types studied.^41−44^ Our results suggest that engineered hLNCs can deliver mRNA to targeted cells and facilitate the transfection of the therapeutic mRNA.

### 3.7 In Vivo Transfection Efficiency

Higher in vivo transfection efficiency of nanoparticles is a critical factor in the success of nanoparticle-based gene therapies. For mRNA therapeutics, achieving high transfection efficiency is essential to introduce specific proteins into target cells and tissues, leading to the desired therapeutic effect.^45^ After obtaining promising in vitro transfection efficiency of hLNCs, we evaluated the nanoparticles for in vivo transfection of FLuc mRNA.

**Figure 6:**
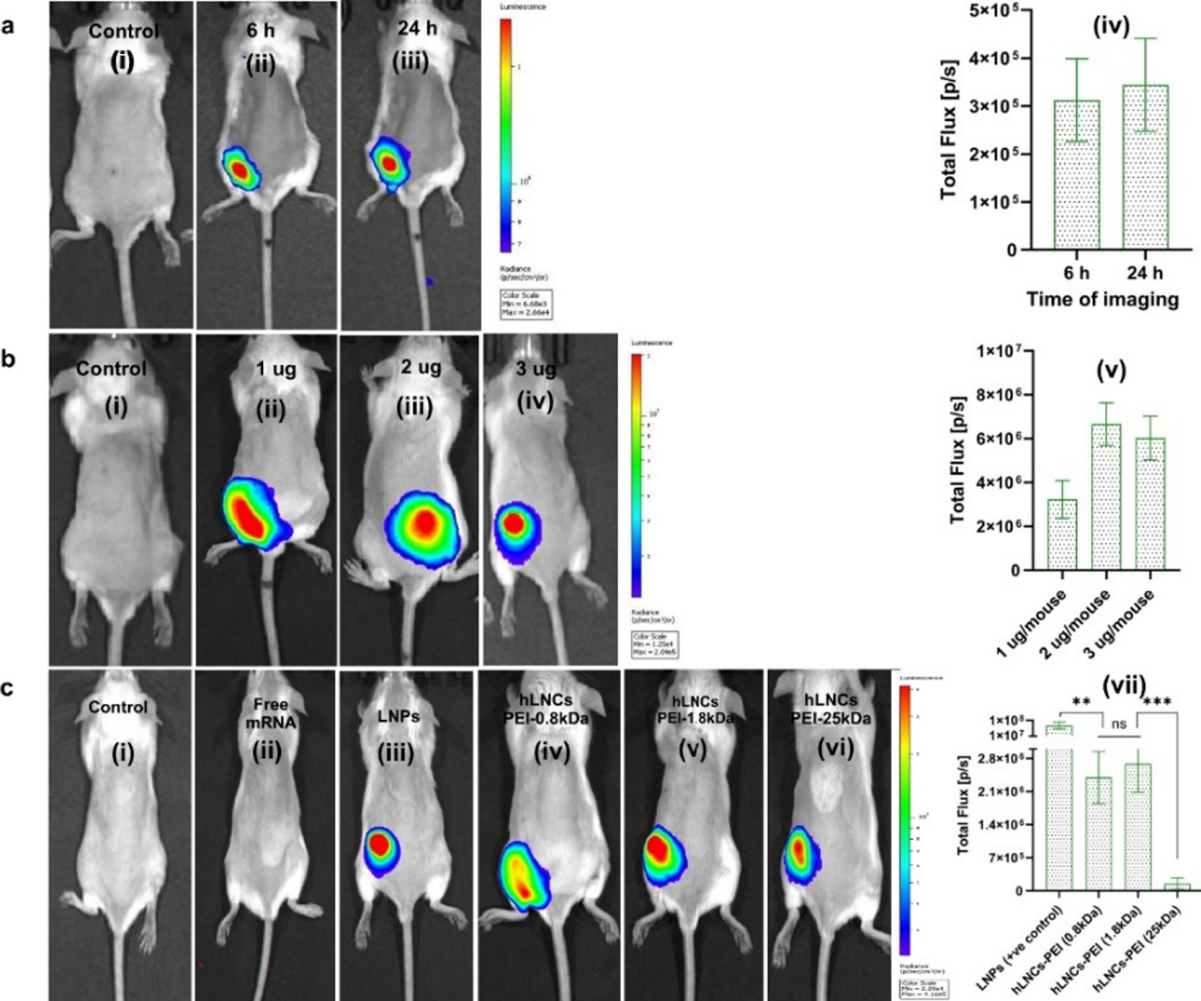
In vivo transfection of mRNA-hLNCs (a) optimization of getting optimum luminescence signal after 6 and 24 h injection of mRNA-hLNCs-PEI (0.8 kDa). (ai–aiv) Representative images of a control mouse, mouse imaged after 6 h, mouse imaged after 24 h, and a graph comparing signals, respectively. (b) Optimization of mRNA dose, (bi–bv) representative images of mouse injected with saline, 1, 2, 3 μg of mRNA-hLNCs-PEI (0.8 kDa) and graph comparing signals, respectively. (c) Transfection of free mRNA or using the LNP/different hLNC system. (ci–cvii) Representative images of a control mouse, injection of free mRNA, mRNA transfected using LNPs, hLNCs-PEI (0.8), hLNCs-PEI (1.8), and hLNCs-PEI (25 kDa), and a graph comparing signal, respectively. 1.5 μg of mRNA was injected/mouse and luminescence signals were collected after 24 h (mice per group= 5).

First, we optimized the time for maximum bioluminescence signals followed by dose optimization by IM injecting mRNA-hLNCs-PEI (0.8 kDa). We observed that the luciferase signals peaked at 6 h after injection (Figure 6aii) and that there was no significant improvement in the luciferase signal after 24 h (Figure 6a(iii, iv)). To optimize the dose, we injected 1, 2, and 3 μg of mRNA-hLNCs (equivalent to mRNA), and measured bioluminescence after 24 h. We observed that the bioluminescence signal in the mice injected with 2 and 3 μg of mRNA-hLNCs is significantly higher than 1 μg (Figure 6b(ii–v)). We note that one of the mice, 3 μg-dosed, showed transfection away from the injection site. We regard this as an outlier and suspect that it was due to the nicking of a vein on injection. Based on the similar transfection patterns (apart from the one aberrant case) and the fact that maximum luminescence intensity was reached with 2 μg, we chose to complex 1.5 μg of FLuc mRNA with hLNCs-PEI (0.8), hLNCs-PEI (1.8), and hLNCs-PEI (25 kDa), and administered IM, collecting the bioluminescence signal after 24 h. LNPs and free mRNA were also injected with an equivalent amount of mRNA via I.M. as a positive and negative control, respectively, and a bioluminescence signal was collected after 24 h. Like in vitro transfection, hLNCs-PEI (0.8 kDa) and hLNCs-PEI (1.8 kDa) showed significantly higher transfection efficiency compared to hLNCs-PEI (25 kDa) (Figure 6c(iv–vii)). Unlike in vitro transfection, hLNCs-PEI (0.8 and 1.8 kDa) showed significantly lower transfection in vivo compared to LNPs (Figure 6c(vii)) but sufficient for mRNA delivery. However, the stability of LNPs was evaluated, and it was found that LNPs are not stable in sugar glass as size increased over 3.0 μm (Supplementary Figure S9). Hence, hLNCs have an advantage over LNPs in terms of robustness. As expected, we did not observe any signal from mice injected with free mRNA. We have thus demonstrated that this new class of hLNCs can be used in an in vivo setting for successful transfection of mRNA.

## 4 Conclusions

In this study, we developed a family of hybrid lipid nanocapsules (hLNCs) optimized for mRNA delivery. Achieving an ideal mRNA:hLNC ratio of 1:200, 1:25/50, and 1:15 for hLNCs-PEI (0.8), hLNCs-PEI (1.8), and hLNCs-PEI (25 kDa), respectively, we demonstrated effective in vitro and in vivo transfection. In vitro transfection of hLNCs (0.8 and 1.8 kDa) is significantly higher than commercial LNPs. We demonstrate that the hLNCs have many features desirable for a robust mRNA delivery nanoplatform. They are synthesized in a simple and gentle aqueous process that yields particles in a narrow size distribution (PDI < 0.2) with an average particle size of ∼40 nm, which is in an ideal range for delivery. We further show that the PDI remains small over a wide pH range and in a wide range of media, suggesting that this particle architecture is likely to be amenable to many manufacturing and delivery scenarios. Additionally, we show that we can dry the hLNCs in the presence of sugar to form a sugar glass, and when they are rehydrated, the small PDI is recovered, and the transfection functionality of mRNA condensed on the particles is fully retained. This strongly indicates that this family of delivery vehicles could be formulated for long-term stability at ambient, and possibly superambient temperatures.

The particles we have demonstrated seem to have an acceptable balance between stability for manufacturing and handling and lability for the eventual release of the mRNA payload. Furthermore, the hybrid structure of lipid and polymer is flexible with respect to charge density, so is likely to be suitable for the delivery of other nucleic acids including pDNA, mRNA, siRNA, etc.

## Supporting Information Available

1H NMR spectra details; DSC spectra; particle size; and zeta potential (PDF)
