# Supplementary material for: Hybrid Lipid Nanocapsules: A Robust Platform for mRNA Delivery
Source: ACS Appl Mater Interfaces. 2024 Mar 20;16(13):15981–92. doi: 10.1021/acsami.4c00992 (PMC10995897; doi:10.1021/acsami.4c00992)
Supplement: Supplementary file 1 — am4c00992_si_001.pdf [file am4c00992_si_001.pdf]

# Supporting Information

## Hybrid lipid nanocapsules: a robust platform for mRNA delivery

Sunil Kumar Yadava[1], B. Pradeep K. Reddy[2], Mark R. Prausnitz[2], and Marcus T. Cicerone[1]*

[1]School of Chemistry and Biochemistry, Georgia Institute of Technology, Atlanta GA, USA

[2]School of Chemical and Biomolecular Engineering, Georgia Institute of Technology, Atlanta GA, USA

*** Corresponds to:**

**Dr. Marcus T. Cicerone**

Email- cicerone@gatech.edu

Phone- (404) 894-2761

**Method:**

**Preparation of lipid nanoparticles (LNPs):** Lipid Nanoparticles were prepared using a microfluidic device by mixing aqueous and organic phases at 1:3 flow rates. Briefly, the aqueous phase was prepared by diluting mRNA in 10 mM citrate buffer, and the organic phase was prepared by solubilizing SM-102 (heptadecan-9-yl 8-[2-hydroxyethyl-(6-oxo-6-undecoxyhexyl)amino]octanoate), (obtained from Broad Pharma.) DMG-PEG2000 (1,2-dimyristoyl-rac-glycero-3-methoxypolyethylene glycol-2000), DSPC (1,2-distearoyl-sn-glycero-3-phosphocholine) (obtained from Avanti Polar Lipids, USA) and cholesterol (obtained from Sigma Aldrich, USA) in molar ratio 50:10:38.5:1.5 in 100% ethanol. The ethanol was removed by dialysis.

**Differential scanning calorimetry (DSC):** DSC of hybrid lipid nanocapsules and its ingredients; Labrafac lipophile WL1349, Kolliphore HS 15, DOPE, and OA-PEI conjugate was performed using DSC250 (Discovery, TA Instruments, USA). The required sample amount was filled into an aluminum pan and sealed. Subsequently, DSC was performed with a 5 °C/min scanning rate from –50 to 120 °C.

**Table: S1**

| | **Trehalose** | **Sucrose** | **Stachyose** | **PVA/Tricine** | **Glycerol** |
|---|---|---|---|---|---|
| **Sugar Glass 1** | √ | - | - | √ | √ |
| **Sugar Glass 2** | - | √ | - | √ | √ |
| **Sugar Glass 3** | - | - | √ | √ | √ |

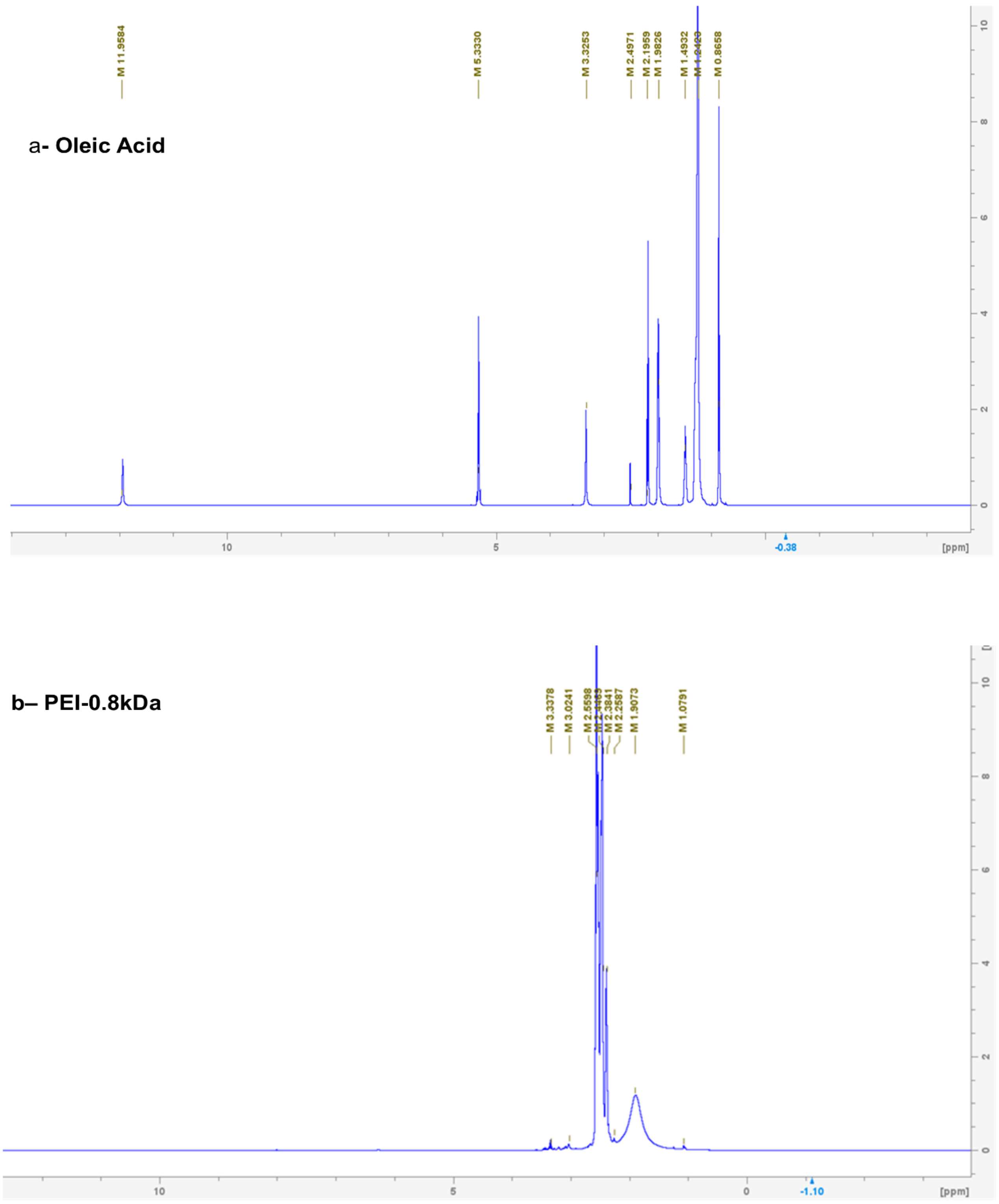


**Figure. S1** $^{1}$H NMR spectra of (a) oleic acid and (b) PEI-0.8kDa (Mw ~800) in DMSO.

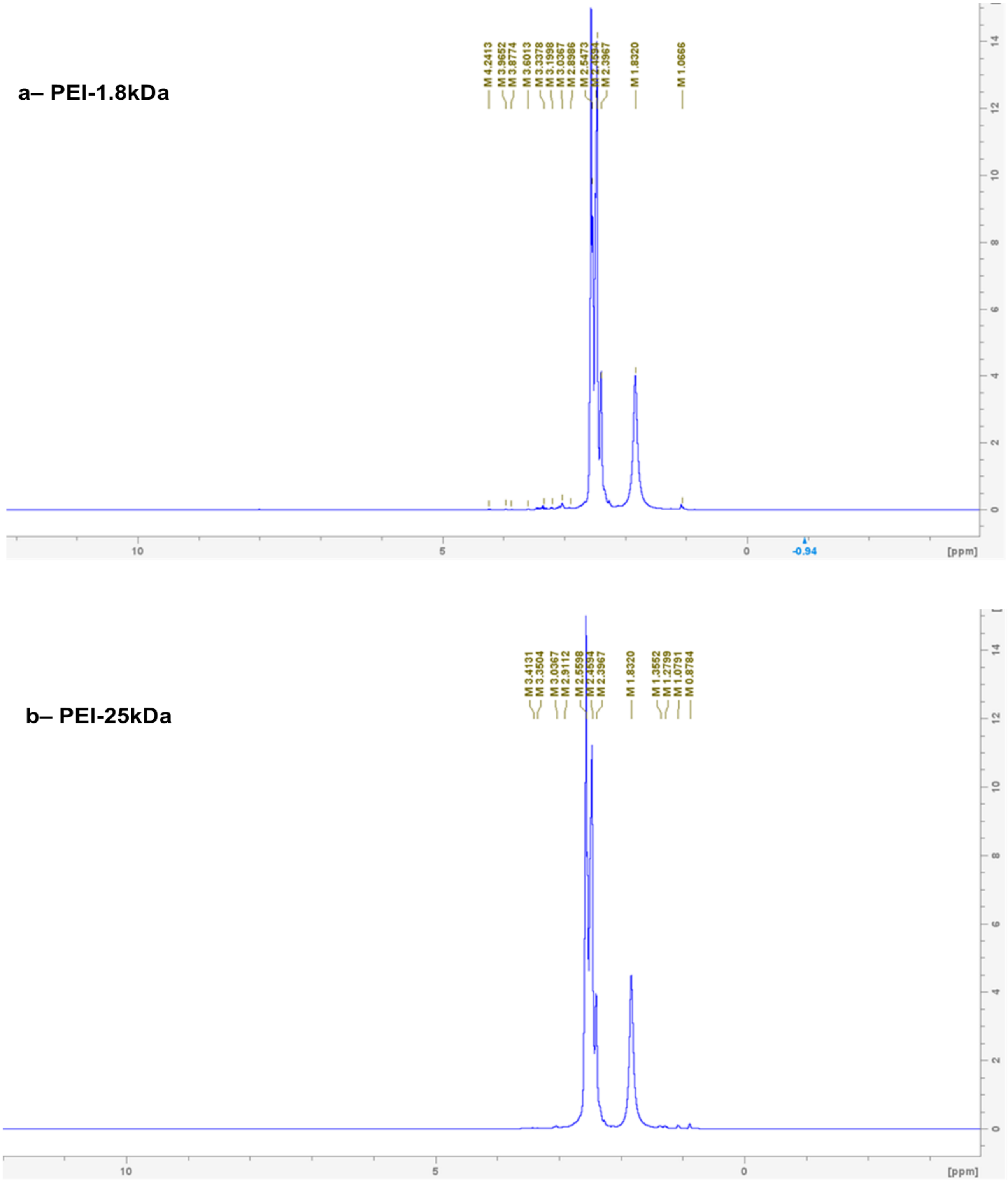


**Figure. S2** $^{1}H$ NMR spectra of (a) PEI-1.8kDa (Mw ~1800) and (b) PEI-25kDa (Mw ~25000) in DMSO.

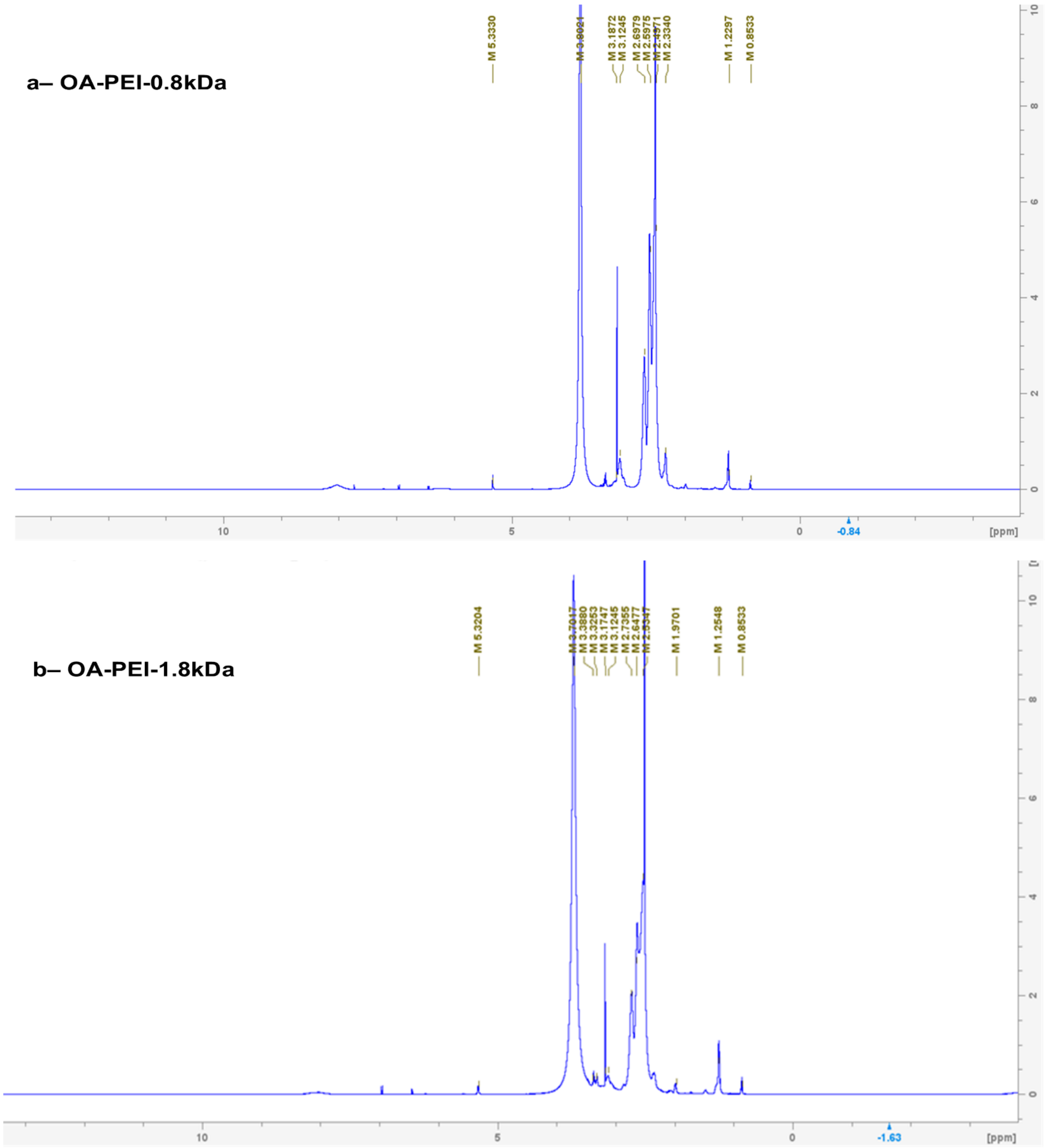


**Figure. S3** $^{1}$H NMR of (a) Oleic acid-PEI-0.8kDa conjugate and (b) Oleic acid-PEI-1.8kDa conjugate in DMSO. The result indicated that Oleic acid-PEI has been successfully conjugated.

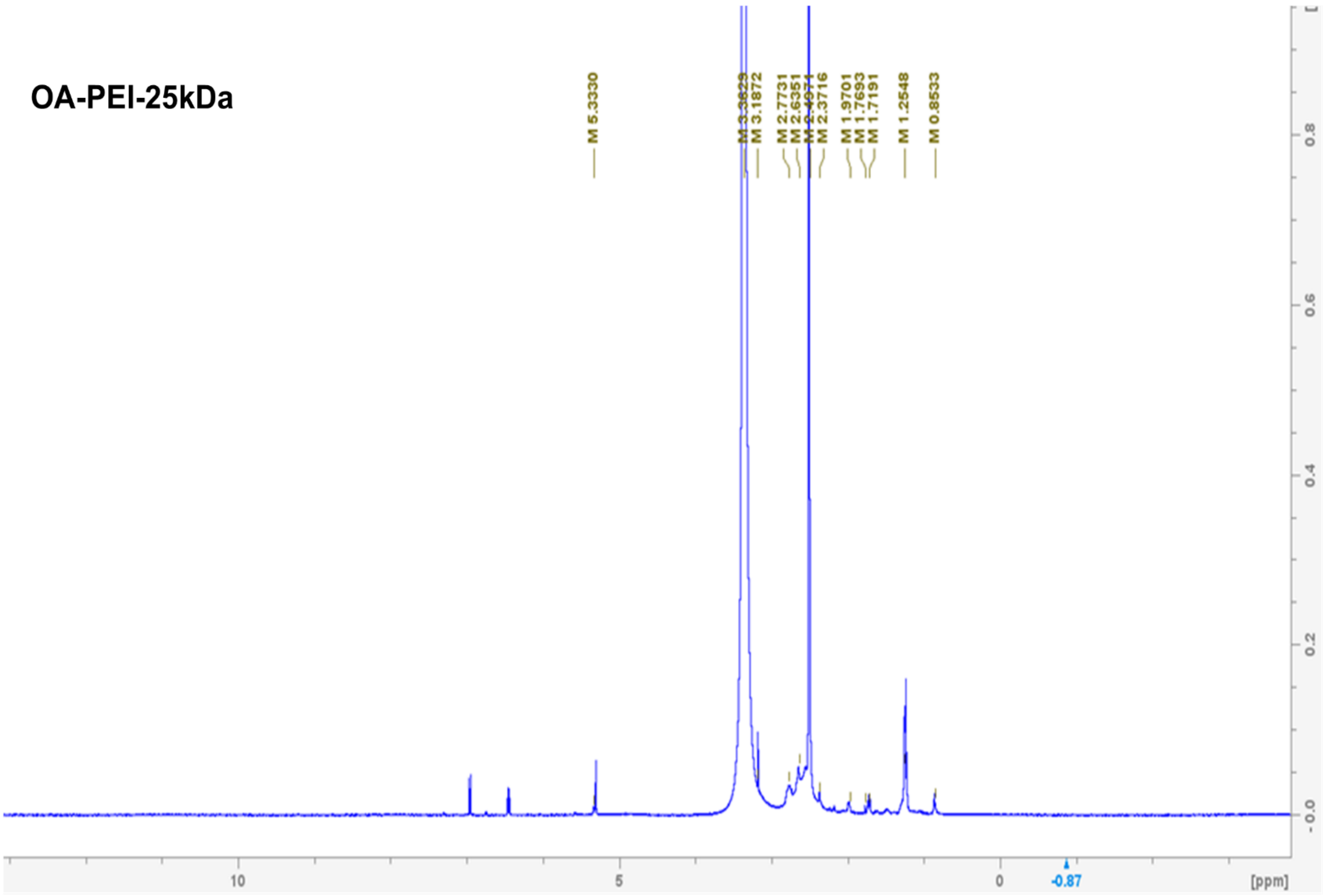


**Figure. S4** $^{1}H$ NMR of Oleic acid-PEI-25kDa conjugate in DMSO. The result indicated that Oleic acid-PEI has been successfully conjugated.

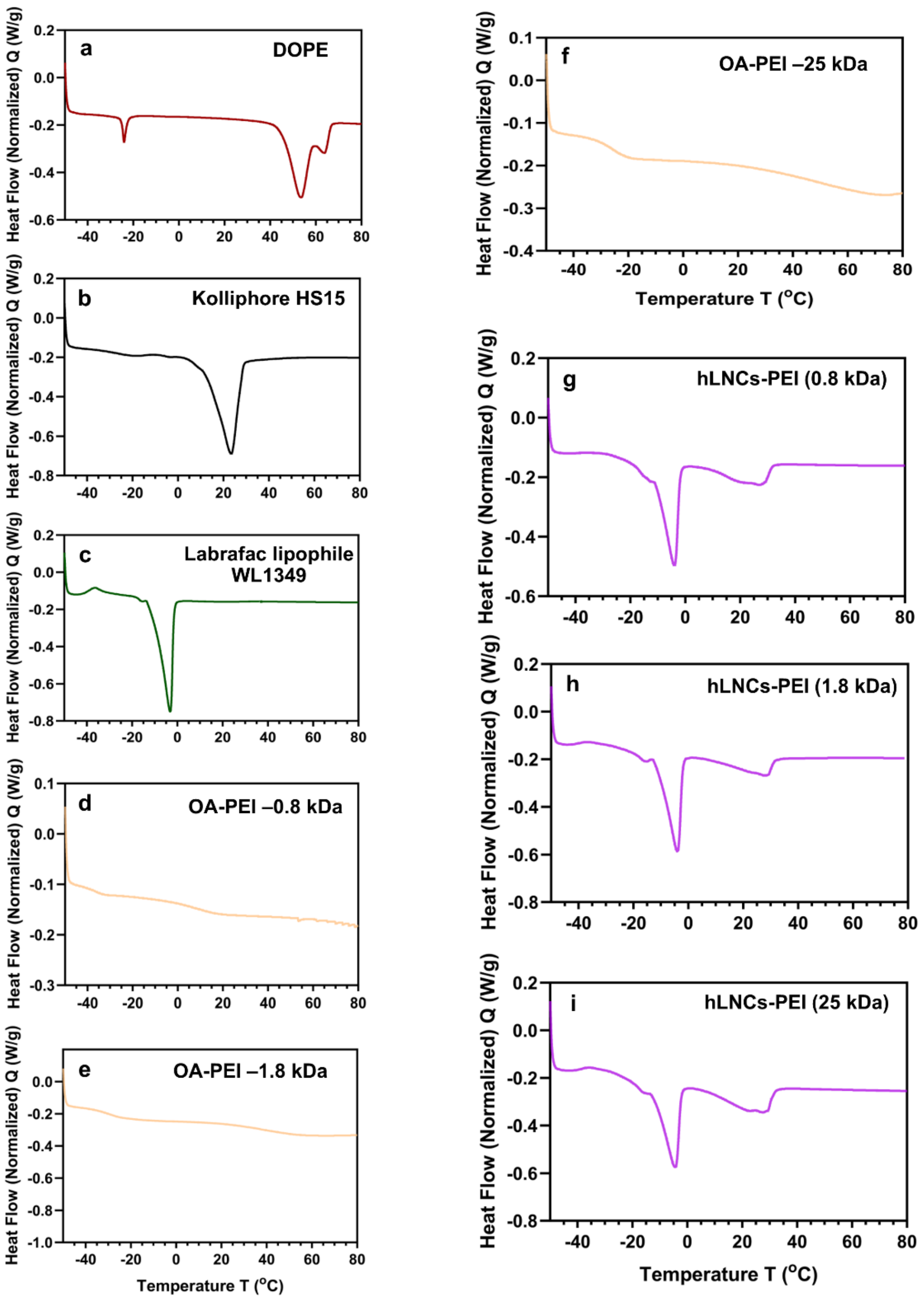


**Figure. S5.** Differential scanning calorimetry spectra of hLNCs and their components. DSC spectra of (a) DOPE, (b) Kolliphore HS15, (c) Labrafac lipophile WL1349, (d) OA-PEI-0.8kDa conjugate, (e) OA-PEI-1.8kDa conjugate, (f) OA-PEI-25kDa conjugate, (g) hLNCs-PEI-0.8kDa, hLNCs-PEI-1.8kDa and hLNCs-PEI-25kDa.

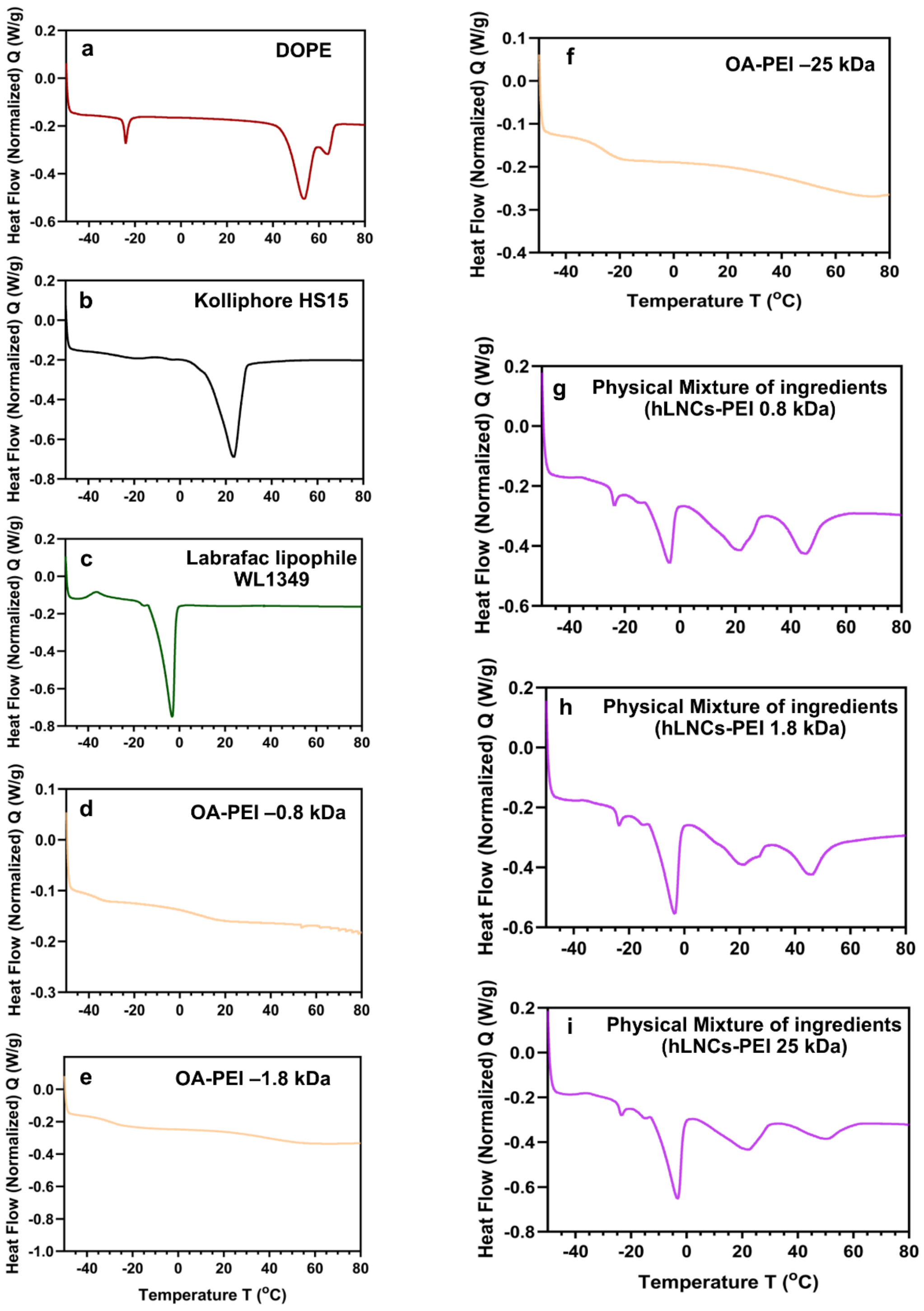


**Figure. S6.** Differential scanning calorimetry spectra of hLNCs components and their physical mixtures. DSC spectra of (a) DOPE, (b) Kolliphore HS15, (c) Labrafac lipophile WL1349, (d) OA-PEI-0.8kDa conjugate, (e) OA-PEI-1.8kDa conjugate, (f) OA-PEI-25kDa conjugate, (g) physical mixture of components (hLNCs-PEI-0.8kDa), (h) physical mixture of components (hLNCs-PEI-1.8kDa) and (i) physical mixture of components (hLNCs-PEI-25kDa).

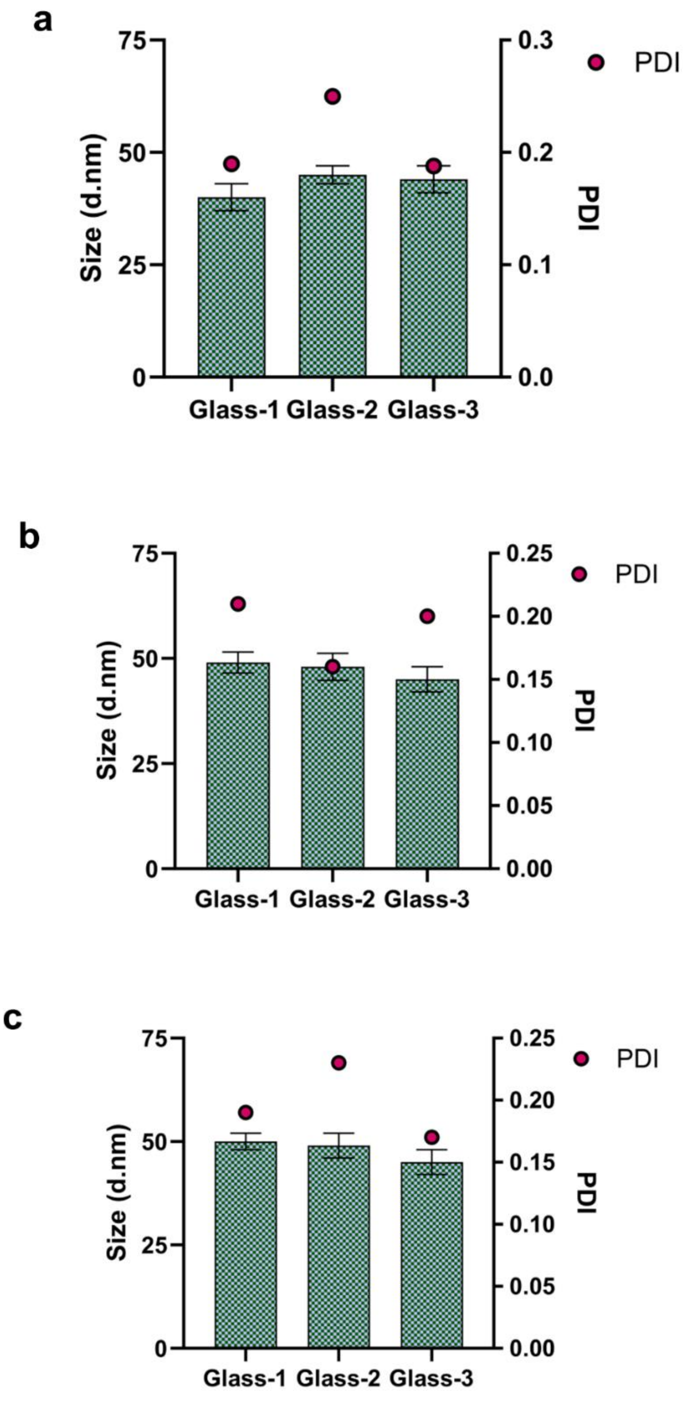


**Figure. S7** particle size of hLNCs measures after dissolving sugar glasses. (a) particles size of hLNCs-PEI (0.8kDa) (b) particles size of hLNCs-PEI (1.8kDa) and (c) particles size of hLNCs-PEI (25kDa). Glass-1 is composed of trehalose + PVA+ Glycerol, Glass-2 is composed of sucrose + PVA+ Glycerol and Glass-3 is composed of stachyose + PVA+ Glycerol.

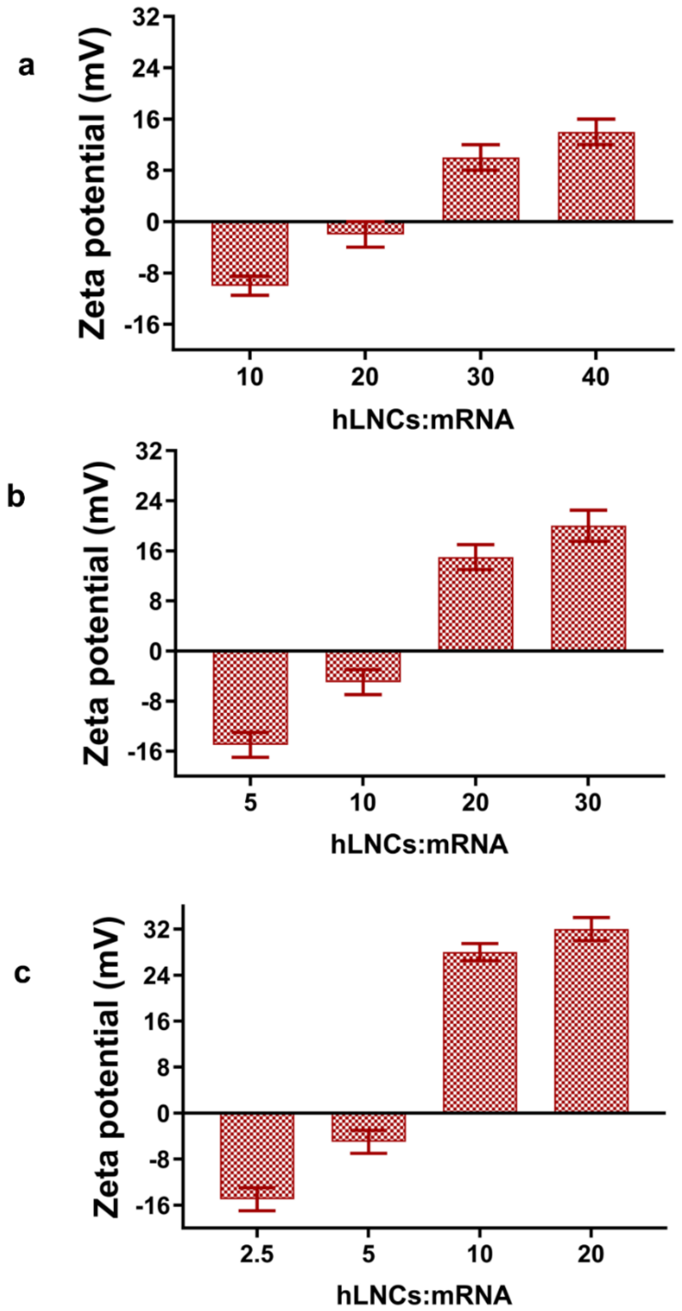


**Figure. S8** Zeta potential of suspension containing a complex of different ratios of hLNCs: mRNA. (a) Zeta potential of mRNA-hLNCs-PEI (0.8kDa) (b) Zeta potential of mRNA-hLNCs-PEI (1.8kDa) and Zeta potential of mRNA-hLNCs-PEI (25kDa).

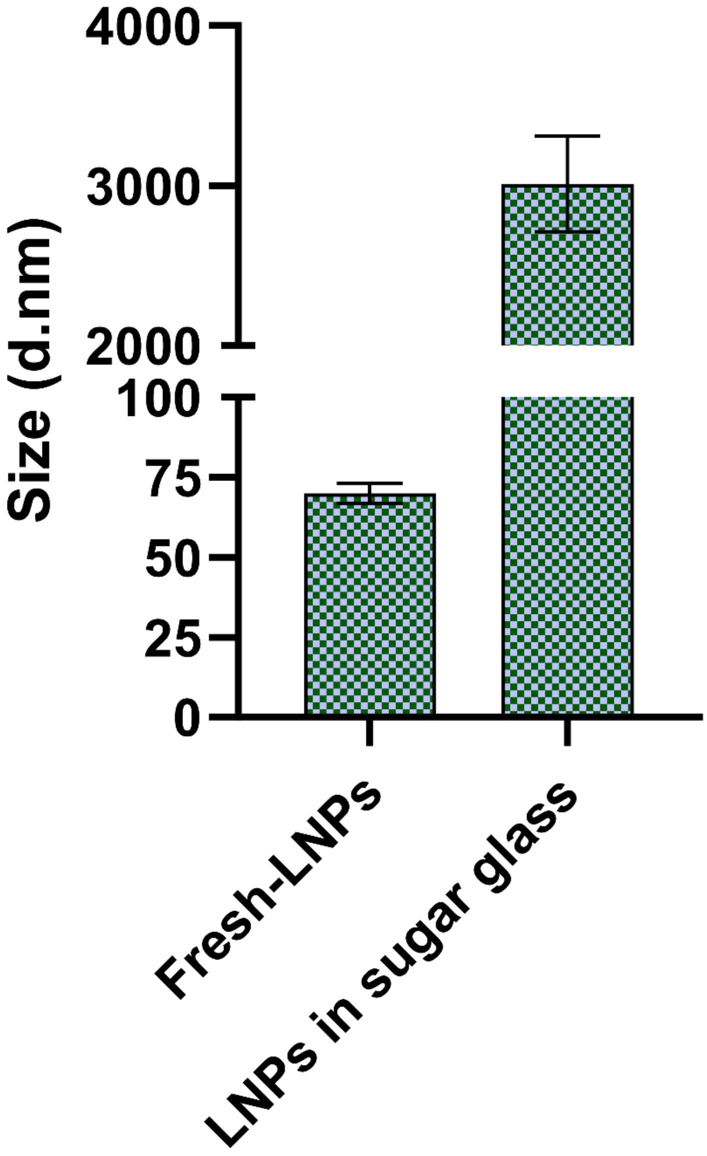


**Figure. S9** Particle size of freshly prepared lipid nanocapsules and size after incorporation into sugar glass.
